# Spine Pseudogout Following Breast Cancer Treatment: A Report of Two Cases

**DOI:** 10.7759/cureus.70198

**Published:** 2024-09-25

**Authors:** Nicholas L Todd, Amber McDermott, Alexander Isla, Frederick P Korpi, Jeffrey Cochran

**Affiliations:** 1 Orthopedic Surgery, Aultman Hospital, Canton, USA; 2 Orthopedic Trauma Surgery, Aultman Hospital, Canton, USA; 3 Orthopedic Spine Surgery, Aultman Hospital, Canton, USA

**Keywords:** breast cancer, calcium pyrophosphate disease, pseudogout, spine pseudogout, spine surgery

## Abstract

Calcium pyrophosphate disease (CPPD) is a commonly diagnosed crystal-induced disease that typically presents as acute monoarticular or oligoarticular arthritis. It is less commonly seen in the spine, and its clinical importance in this area is still relatively understudied. Isolated spinal CPPD is quite rare; a diagnosis of spinal CPPD is almost always accompanied by peripheral CPPD. We present two patients who were both being treated for breast cancer. They underwent spine surgery and were subsequently diagnosed with isolated CPPD of the spine by pathology specimens. Neither patient had a history of CPPD prior to the surgery. We hypothesize there is a potential link between breast cancer and its treatment and isolated spinal CPPD; however, more research and case reports are needed to begin making conclusions and understand the relevance.

## Introduction

Crystal-induced arthropathies are a group of metabolic diseases that are characterized by crystal deposition into tissue [1]. Calcium pyrophosphate disease (CPPD, pseudogout) is a crystal-induced arthropathy caused by the deposition of calcium pyrophosphate (CPP) crystals. CPPD generally affects the knees, wrists, metacarpophalangeal (MCP) joints, hips, shoulders, and ankles [2]. Spinal pseudogout can present with acute or chronic symptoms, often mimicking other spinal disorders such as degenerative disc disease, ankylosing spondylitis, or infectious spondylodiscitis, leading to diagnostic challenges. The diagnosis is frequently delayed, with imaging studies playing a role in identifying calcifications within the intervertebral discs, ligaments, and synovial joints that may be suggestive of spinal CPPD [3]. These changes seen on imaging can be non-specific for pseudogout of the spine. Definitive diagnosis can require pathology specimens. The clinical importance of CPPD in the spine remains unclear currently due to a lack of research on its natural history.

The exact pathogenesis of spinal CPPD remains poorly understood, but it is thought to be associated with age-related cartilage degeneration, metabolic abnormalities, and genetic predispositions. Factors such as osteoarthritis, trauma, and underlying metabolic disorders, including hyperparathyroidism and hemochromatosis, have been implicated in the development of CPPD [3]. We present two patients who were both undergoing treatment for breast cancer. They were subsequently diagnosed with isolated spinal CPPD by pathology specimens after spine surgery. Currently, no literature demonstrates a correlation between cancer and the development of pseudogout. Additionally, pseudogout is not known to be induced by any medications currently.

## Case presentation

Case 1

We present the case of a 72-year-old female with a past medical history significant for arthritis and metastatic breast cancer that was HER2/Neu positive. She was undergoing chemotherapy with denosumab, vinorelbine tartrate, and trastuzumab. Her latest bone scans were reported to be stable, according to her oncologist.

She presented to the orthopedic spine clinic with low back pain and right hip pain. The pain was a sharp and constant pain in her right hip and buttock that radiated down her right thigh to the knee. The physical examination revealed tenderness to palpation over the right sacrum, right gluteal area, right greater trochanter, and iliotibial band. Forward flexion of the lumbar spine to 90 degrees and 10 degrees of hyperextension elicited pain in the right gluteal region. The straight leg raise test performed bilaterally was negative. The patient was able to toe- and heel-walk without weakness and had a normal gait. She demonstrated hypersensitivity to the lower legs bilaterally in a non-dermatomal distribution. Lumbar myotome testing demonstrated no deficits. The patellar and Achilles reflexes were absent bilaterally. No evidence of bilateral ankle clonus was observed.

Plain radiographs of the lumbar spine revealed advanced degenerative disease at the L5-S1 level and milder degenerative disease throughout the remainder of the spine. There was also a Grade 1 spondylolisthesis at the L3-L4 level, which was stable. After an unsuccessful trial of non-surgical treatment, including nonsteroidal anti-inflammatory drugs (NSAIDs) and physical therapy, an MRI of the lumbar spine was obtained.

**Figure 1:**
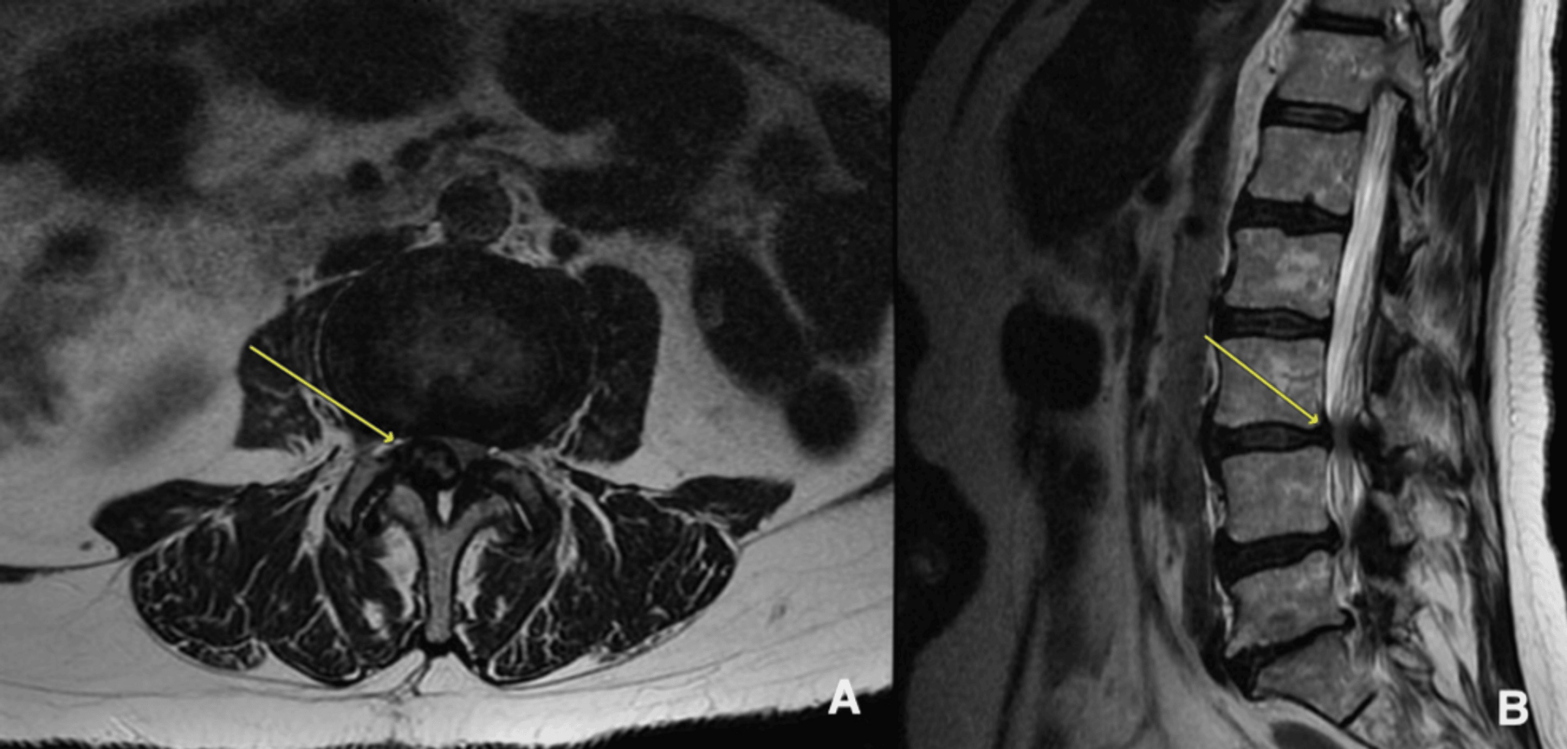
MRI of the lumbar spine demonstrating a facet cyst at L3-L4 (yellow arrows). (A) axial view; (B) sagittal view

The MRI of the lumbar spine showed advanced degenerative changes at the L5-S1 level with facet arthrosis and mild foraminal stenosis on the left. At L4-L5, there was facet hypertrophy and ligamentum flavum hypertrophy, causing mild lateral recess stenosis. At the L3-L4 level, ligamentum flavum hypertrophy and a large, right-sided, complex facet cyst resulted in severe spinal stenosis, as demonstrated in Figure 1. A lumbar MRI performed 26 months earlier showed only mild to moderate spinal stenosis at L3-L4. There was no evidence of metastatic disease in either study.

With progressive symptoms, failed non-surgical treatment, and a positive correlation of the symptoms with the MRI findings, surgical intervention was offered. Her symptoms were predominantly radicular in nature, and the L3-L4 spondylolisthesis was stable. Considering these factors, the patient's complicated medical history, and the significant morbidity and prolonged recovery associated with lumbar fusion, it was determined that micro-decompression at L3-L4 and L4-L5 would be the best option to provide long-term improvement.

The patient was taken to the operating room for routine micro-decompression at L3-4 and L4-L5. A partial L3-L4 laminectomy was completed bilaterally. The ligamentum flavum was removed on the left, and dissection was carried out across the midline to the right side, where a large epidural compressive lesion was found. This correlated with the MRI findings. The L3 laminectomy was extended in order to gain better access to the entire mass. During removal, it was determined that the consistency of the mass was not typical of ligamentum flavum or facet capsule. It was found to emanate from the facet joint and was adherent to the dura. The mass was dissected off of the dura and facet joint, resulting in decompression of the neural structures. A small amount of lesion was left on the dura to avoid dural injury. A routine decompressive partial laminectomy was performed at the L4-L5 level. Due to the atypical appearance of the lesion at L3-L4, it was submitted for evaluation by the pathology department. The report of the histological examination revealed portions of fibrotic tissue with extensive CPP deposition. The submitted specimen was negative for malignancy.

Case 2

The second case in our series is a 52-year-old female who was initially diagnosed with breast cancer four years prior to presentation to the spine clinic. The cancer was characterized as oligometastatic cancer of the left breast that was ER positive and HER2/Neu negative. She had been treated with abemaciclib, letrozole, leuprolide, and denosumab. Her regimen had been unchanged to date. Bone metastasis was first detected two months after her initial diagnosis and remained isolated to her right ninth rib. It was determined to be metastasis by biopsy.

The patient first presented to the orthopedic spine clinic three years following her initial diagnosis of breast cancer. At the initial visit, the patient reported back pain that first began one year prior to her presentation. There was no inciting injury. The pain was described as an achiness and pain that radiated from her right lower back into her right gluteal region to her right knee. She also complained of right-sided lower back spasms that were occasionally a 10/10 in terms of pain. She had previously been given an epidural injection with minimal relief. Oral pain medications did not relieve her pain. The spine exam demonstrated a positive straight leg test on the right, with normal strength and sensation in the lower extremities. Deep tendon reflexes were intact. No ankle clonus was detected.

**Figure 2:**
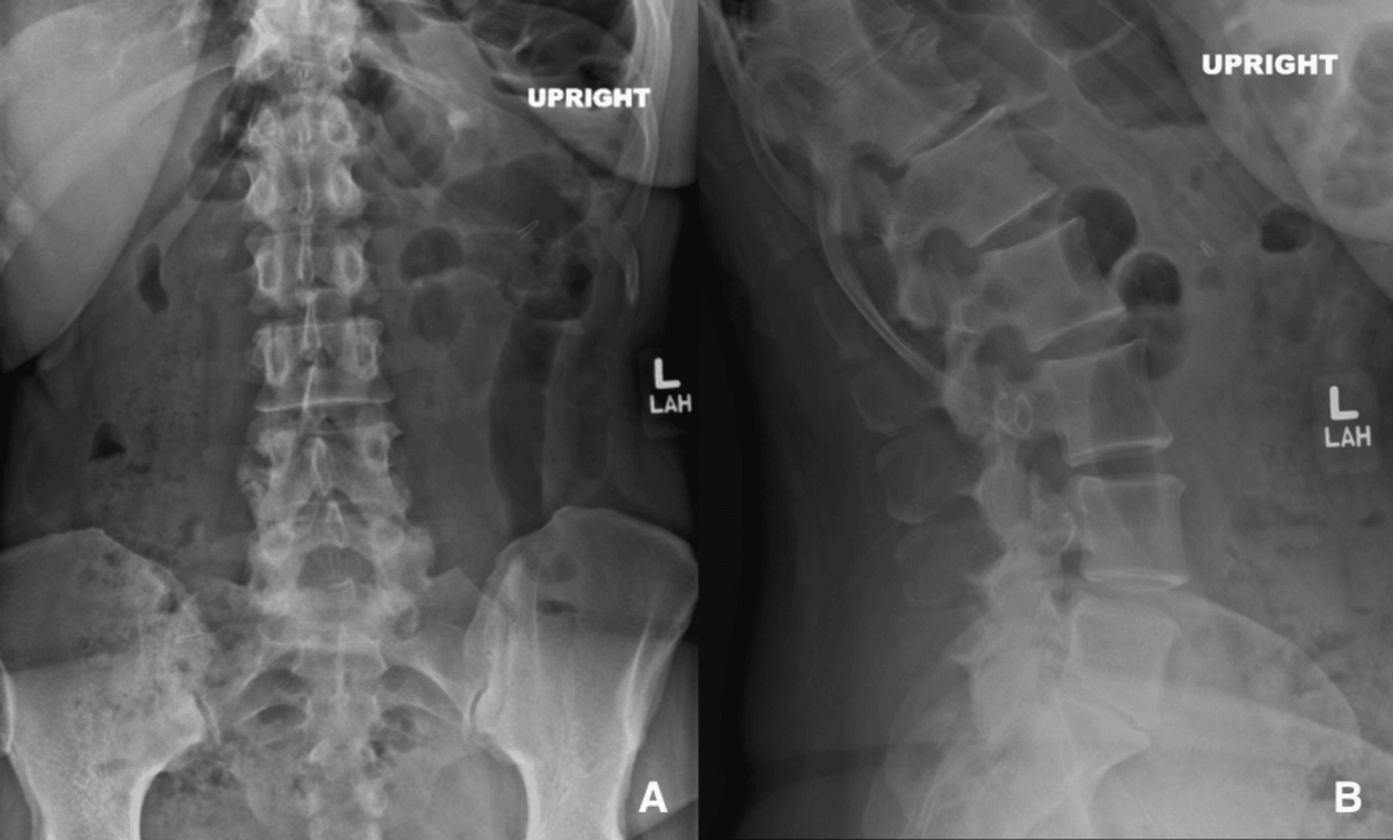
Radiographs of the lumbar spine. (A) anterior-posterior view; (B) extension view

**Figure 3:**
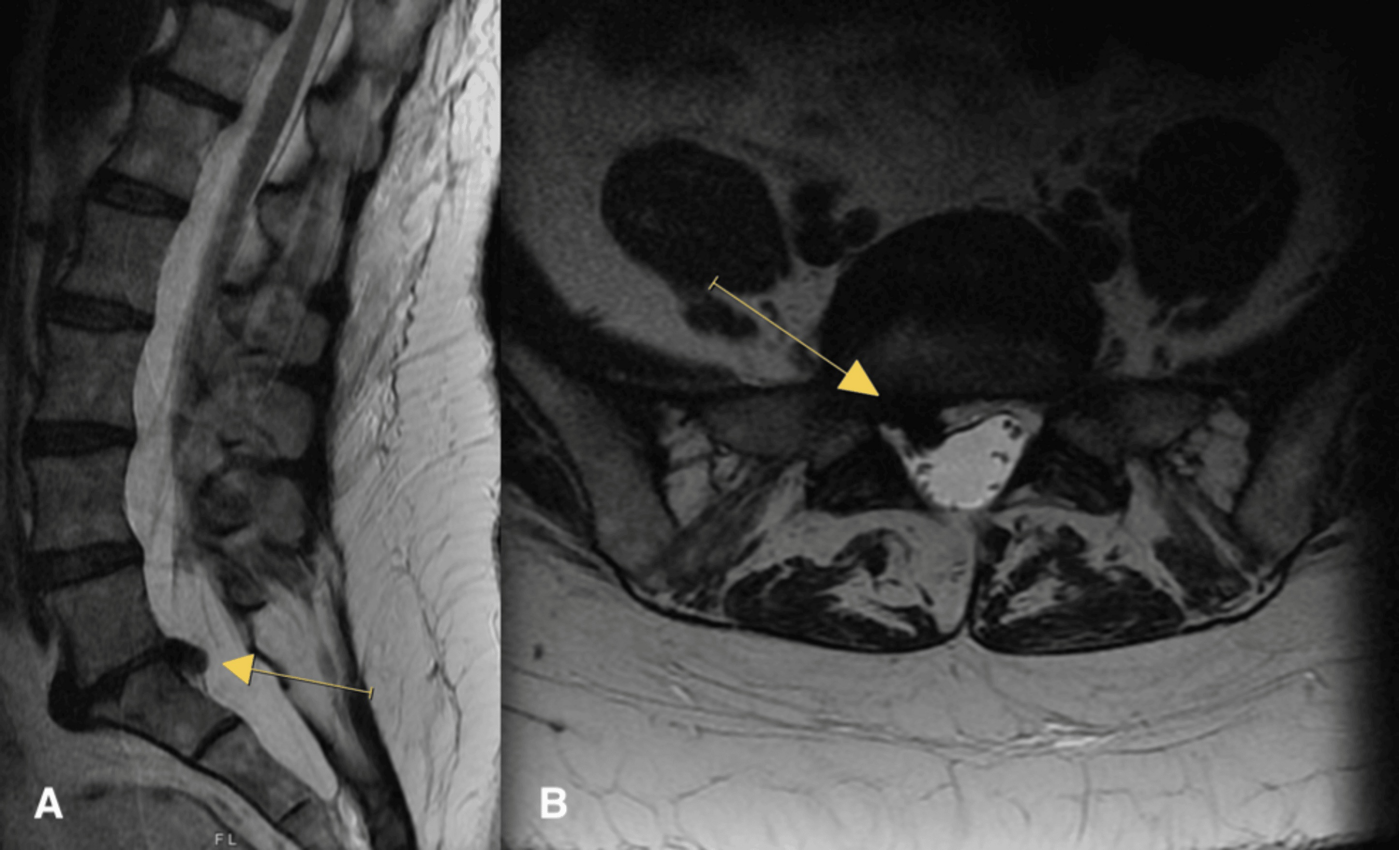
MRI of the lumbar spine demonstrating disc herniation at L5-S1 (yellow arrows). (A) sagittal view; (B) axial view

Lumbar spine X-rays demonstrated mild degenerative changes at L4-L5, L3-L4, and T12-L1. There was advanced degenerative disc disease at L5-S1 with complete collapse of the disc space. There was no instability on dynamic X-rays. These changes are demonstrated in Figure 2. A lumbar MRI performed two months prior to the initial consultation demonstrated L5-S1 disc displacement on the right with caudal migration, as seen in Figure 3.

The MRI findings were consistent with the symptoms and physical examination. She had no improvement with treatment to date, which had included oral analgesics, physical therapy, and lumbar epidural steroid injections. Having failed non-surgical treatment, she elected to proceed with microdiscectomy at L5-S1 on the right.

The surgery proceeded in the usual fashion. Upon opening the spinal canal, we found that the S1 nerve root was displaced significantly by an epidural mass. As we removed this mass, we observed a chalky consistency typically seen with gouty tophus. Fragments of this mass were submitted as specimens separate from the disc material that was submitted. At the conclusion of the procedure, the L5 and S1 nerve roots were noted to be completely decompressed. The pathology report indicated that the epidural mass was fibrocartilaginous with extensive calcification and foci of CPP deposition. This was consistent with the diagnosis of pseudogout.

## Discussion

Spinal CPPD is generally uncommon. One study described that 15% of patients with CPPD also had CPP in their spine when undergoing anterior interbody fusions [4]. Another case series demonstrated that 25% of patients with CPPD had spinal CPPD as well and noted those with spine involvement had widespread peripheral disease [5]. Isolated spinal CPPD is assumed to be quite rare, and there is currently no literature describing the incidence of isolated spinal CPPD. According to the present literature, CPPD of the spine is almost always associated with peripheral disease. In our patients, neither had a history of peripheral CPPD.

We explored the possible connections between CPPD, breast cancer, and chemotherapy. We speculate that the chemotherapy regimens that the patients were on precipitated the deposition of CPP in the spine. The only medication that both presented patients were taking was denosumab. The present literature does not demonstrate a correlation between CPPD and denosumab. We speculate that an unstudied side effect of the medication is CPPD. The only current well-described and studied side effects of denosumab are osteonecrosis of the jaw, back pain, hypocalcemia, and hypophosphatemia. Some of the current indications for the use of denosumab include osteoporosis in postmenopausal women with high fracture risk and breast cancer patients on aromatase inhibitors with metastatic disease to the bone. Denosumab has been studied in the breast cancer population with no current correlation to CPPD.

The pathogenesis of CPPD involves abnormal pyrophosphate metabolism, resulting in increased extracellular pyrophosphate levels, which combine with calcium to form CPP crystals. These crystals deposit in cartilage and synovium, triggering an immune response that includes the activation of the NLRP3 inflammasome, leading to the release of pro-inflammatory cytokines, particularly IL-1β [6]. This results in acute joint inflammation, resembling gout, but caused by CPP crystals rather than uric acid crystals. Over time, chronic inflammation can lead to joint degeneration and arthritis.

Additionally, cancerous processes are known to produce significant stress on the body. Specifically, breast cancer can cause general bodily inflammation through multiple mechanisms. As tumors grow, they release pro-inflammatory cytokines and chemokines, such as IL-6, IL-1β, and TNF-α, which promote systemic inflammation [7]. The presence of circulating tumor cells can activate immune cells, such as macrophages and neutrophils, contributing to chronic inflammation. Furthermore, cancer treatments such as chemotherapy and radiation can induce an inflammatory response as they damage healthy tissues, which leads to the release of inflammatory mediators [7]. There may be a connection between the general pro-inflammatory state of breast cancer and the development of CPPD; however, there is no literature demonstrating an association.

We hypothesize that the presentation of isolated spinal CPPD in our patients may be an unrecognized or unstudied complication of chemotherapy or breast cancer. At this time, there is no clear association between either possible etiology. Since the incidence of isolated spine CPPD is likely relatively low, we believe that further research is indicated to study a possible connection between these complex pathologies.

## Conclusions

We present two cases of isolated CPPD of the spine, both of which involved patients with a medical history of breast cancer treated with chemotherapy. While this represents a small subset of patients, it underscores the need for further investigation into this rare clinical presentation. Its clinical significance remains unclear except when it results in neurologic compression. A connection between breast cancer, chemotherapy, and CPPD of the spine has not been established. Further research and case reports are needed to understand these findings in the setting of the complex pathophysiology of breast cancer and its treatment. These two cases provide valuable insights that can significantly contribute to advancing understanding in this area and lay a robust foundation for future research.
